# Law and urban governance for health in times of rapid change

**DOI:** 10.1093/heapro/daab064

**Published:** 2021-12-12

**Authors:** Scott Burris, Vivian Lin

**Affiliations:** 1 Beasley School of Law, Temple University, Philadelphia, PA, USA; 2 Faculty of Medicine, the University of Hong Kong, Hong Kong Special Administrative Region, China

**Keywords:** community empowerment, determinants of health, governance, government, health promoting policies, legal epidemiology

## Abstract

Governance is an important factor in urban health, and law is an important element of healthy governance. Law can be an intervention local government wields to influence behavior and shape environments. Law can also be an important *target* of health promotion efforts: Law and the enforcement and implementation behaviors it fosters can promote unhealthy behaviors and environmental conditions, and can act as a barrier to healthy interventions or practices. Finally, law is a design and construction tool for the organization of governance. Law is the means through which cities are formally established. Their powers and duties, organizational structure, boundaries and decision-making procedures are all set by law. Regardless of the form of government, cities have legal levers they can manipulate for health promotion. Cities can use tax authority to influence the price of unhealthy products, or to encourage consumption of healthy foods. Cities can use their legal powers to address incidental legal effects of policies that they themselves cannot control. Cities may also have the authority to use law to address deeper determinants of health. The overall level of income or wealth inequality in a country reflects factors well-beyond a local government’s control, but city government nonetheless has levers to directly and indirectly reduce economic and social inequality and their effects. A renewed focus on law and urban governance is the key to assuring health and well-being and closing the health equity gap.

## INTRODUCTION—WHY LAW AND URBAN GOVERNANCE FOR HEALTH

Fifty-five percent of the world’s population lived in cities as of 2018, a proportion predicted to increase to 68% by 2050 (United Nations Department of Economic and Social Affairs Population Division, 2019). Rapid urbanization, particularly in low- and middle-income countries, has strained the adaptive capacity of city government. Basic infrastructure—sanitation, housing, transportation, schools and healthy amenities—has lagged behind population growth in many places. Housing has been built haphazardly where it can be, without regard to where residents will work, study, shop, worship or exercise. Conditions are worst for those with the fewest resources, creating or exacerbating health inequalities and building poorer health and more limited opportunity into the life course of children.

How communities cope with growth is determined heavily by resource availability and political conditions, including the character of local governance. ‘Governance’ encompasses both the formal organization of management capacity, responsibility and authority within local government and the broader networks of influencers—NGOs, businesses, informal citizen groups—that shape policy decisions and implementation (Burris *et al.*, 2005, 2008). Good governance can help secure healthier housing and living conditions, access to safe water and sanitation, safer working environments and neighborhoods, food security, and access to services such as education, health and transportation (Kjellstrom *et al.*, 2007).

In the last half-century, there has been substantial change around the world in thinking and action about the powers of local governments and the division of authority among citizens, local, regional and national governments. Urban planning, as a field, has embraced systems approaches and collaborative models that recognize most urban planning and management challenges as ‘wicked problems’ that are difficult to solve because of the complex intersecting forces, incommensurable values and feedback loops at play (Rittel and Webber, 1973; Adams, 2011; Innes and Booher, 2018). Both the allocation of authority within government—the traditional organizational chart of police, health, education and so on—and an expanded role for actors outside government have been variables in this on-going experiment in the governance of complex social systems, and research in economics and public management has not found a universally desirable model for allocating authority within local governments or between national, regional and local levels (Plaček *et al.*, 2020).

Some practical insights for local health governance have emerged from more than 30 years of experiences of the healthy cities movement around the world. In 2016, more than 100 mayors came together at the 9th Global Conference on Health Promotion in Shanghai and adopted a consensus statement on healthy cities including five governance principles:

- *Integrate health as a core consideration in all policies*: prioritize policies that create co-benefits between health and other city policies, and engage all relevant actors in partnership-based urban planning;
- *Address all—social, economic and environmental—determinants of health*: implement urban development planning and policies which reduce poverty and inequity, address individual rights, build social capital and social inclusion and promote sustainable urban resource use;
- *Promote strong community engagement*: implement integrated approaches to promoting health in schools, workplaces and other settings; increase health literacy; and harness the knowledge and priorities of our populations through social innovation and interactive technologies;
- *Reorient health and social services towards equity*: Ensure fair access to public services and work towards Universal Health Coverage;
- *Assess and monitor wellbeing, disease burden and health determinants*: use this information to improve both policy and implementation, with a special focus on inequity—and increase transparency accountability (World Health Organization, 2017a).

Law is an important element of healthy governance. Consistent with contemporary practice in socio-legal research, ‘law’ includes f legal texts like constitutions and statutes, but also the formal policies of public and private institutions, the implementation/enforcement practices of legal agents and the beliefs about the law prevailing among those subject to it (Stryker, 2013). In public health and the emerging empirical discipline of legal epidemiology, ‘interventional’ law is legal action intended to influence health behaviors, environments or outcomes directly; ‘infrastructural’ law establishes the powers and duties of institutions and agents in the health or broader governance system; finally, ‘incidental’ law is the much larger body of law that has little or no explicit link to health but nonetheless may influence or mediate other social determinants of health (Burris *et al.*, 2010).

Health policy commentators and the Ottawa Charter point to the importance of healthy public policy within supportive, well-governed environments. Intersectoral governance and health-in-all policies have been promoted for decades as approaches for addressing social determinants of health, but such initiatives can be short lived and dependent on personalities and politics (Lin and Kickbusch, 2017; McQueen *et al.*, 2012).The use of legal instruments, on the other hand, potentially can lock in and sustain structural change in relation to inequitable exposure or unfair processes and thereby contribute to better health outcomes—provided there are good ideas—and political consensus—on what new governance structures and practices should be. The passage of the Public Health and Wellbeing Act in Victoria (Australia; Public Health and Wellbeing Act 2008, 2008), in which the state parliament explicitly required local government to address social determinants of health through intersectoral planning, has been cited as an example of institutionalizing an action framework on social determinants in local government (Lin, 2013).

In recognition of cities being complex and dynamic systems that vary enormously from each other and in a context of on-going scholarly debate about the optimal design of urban governance, this article will consider the effectiveness of law as a practical instrument of health governance and health promotion at the local level in several modes of action. First, law can be a tool for government to influence behavior and shape environments within the municipality, including by shaping governance networks and processes within local institutions. Second and closely related, legal change can address instances in which existing law acts as a barrier to healthy governance practices or otherwise harms health. Finally, law is the primary mechanism for formally establishing the structure of government and the opportunities for participation in formal governance. This role of law provides the greatest opportunity for enduring structural change, but accordingly also demands some level of confidence and consensus on what that change should be. Developing the ‘architecture of the future’ is thus the area of potentially greatest pay-off and greatest on-going difficulty in the fostering of healthy urban governance.

## THE PLACE OF LAW IN URBAN HEALTH PROMOTION

In complex systems like large cities, governance is networked. Governance networks emerge and evolve to manage complex dynamic systems in urban settings. They are built around but not limited to the roles and resources of local government (Burris *et al.*, 2008; Hein, 2003). Local governance, in turn, is nested within regional, national and even international governance networks, which can powerfully shape local conditions but over which locals have little direct control (Burris *et al.*, 2006). Governance networks emerge from and are conditioned on formal legal arrangements. Law defines the powers, duties and jurisdiction of official government entities (Gostin and Wiley, 2016). It also both sets the terms and procedures of private civil society participation in formal government decision-making, and gives private citizens rights, privileges and discretion to act in markets, private institutions and families. In considering urban governance and health, then, law offers a practical, tool-oriented perspective on action to promote health (Burris *et al.*, 2010).

### Law and health promotion in the modern urban setting

Health is significantly determined by the conditions of life where people dwell, learn, work and play (Commission on Social Determinants of Health, 2008). For most of the world’s residents, that is an urban area. All the well-identified health behaviors targeted for health promotion activities play out in cities: smoking and other unhealthy substance use, unhealthy eating, sedentariness. Known unhealthy environmental exposures, from poor sanitation and water access through residential lead and mold to polluted air are a problem for urban residents almost everywhere. The social environment, including behavioral norms and the allocation of the resources necessary for health, is the ultimate determinant of whether the city offers the conditions necessary for health, and so the most important target of health promotion broadly conceived. Law has a well-recognized role in all these levels of health and health promotion.

Law can be used to change individual behavior through measures like clean indoor air and vaccination laws. These specify things individuals must or must not do, but also have an influence on the social and physical environment: drink driving prohibitions do not work simply by deterring an individual from drinking and driving, but also by helping to change social norms about the behavior from tolerant to intolerant. Likewise, laws that limit individual smoking options also serve as reminders of the dangers of smoking and change the social identify of the smoker. Law is also commonly used in the form of industrial regulation to reduce air and water pollution, prevent the sale of dangerous consumer products, and instigate safer road and vehicle designs.

Less frequently recognized is the character of law as a *target* of health promotion efforts, although the mantra of ‘healthy public policy’ (and more recently, concerns about the commercial determinants of health) suggests that legislation and regulation must be core to the health promotion enterprise (Kickbusch *et al.*, 2016; McKee and Stuckler, 2018). Law and the enforcement and implementation behaviors that follow can promote unhealthy behaviors and environmental conditions. For example, restrictive abortion laws, punitive drug laws and laws that limit women’s property rights have all been found to create or exacerbate health risks (Burris *et al.*, 2004; Muchomba *et al.*, 2014; Latt *et al.*, 2019). Both negative and positive effects highlight the place of law in a social determinants perspective: tax, labor and education laws all play a powerful role in shaping the distribution of goods necessary for health, and are all therefore important targets of efforts to create healthy places (Komro *et al.*, 2016; Markowitz *et al.*, 2017; Montez *et al.*, 2019; 2020; Van Dyke *et al.*, 2018). Indeed, it is fair to say that any health promotion activities that aim to move beyond proximal causes must address the ‘legal determinants’ of health (Gostin *et al.*, 2019).

Finally, law is a design and construction tool for the organization of governance. Law is the means through which cities are formally established. Their powers and duties, organizational structure, boundaries and decision-making procedures are all set by law. In this, law also sets the rights, powers and duties of individuals and non-governmental organizations. The importance and potential for action in this aspect of law cannot be understated (Montez, 2017). When we work on improving health systems, we are to a considerable extent engaged with systems set up and maintained by law. Law will establish ownership and regulation of local utilities. Law sets boundaries and oversight of transportation systems, planning boards, school districts and economic development authorities. When we talk about whole-of-government or health-in-all policies approaches, we are using vocabularies of cooperation and health that may obscure the fact that a problem like getting police to work more closely with public health and health care agencies in managing acute mental illness arises in significant part because of prior legal decisions about what kind of agencies to create to deal with public health and safety (Wood and Beierschmitt, 2014).

### The legal powers and reach of local government—and their limitations

Apart from the rare city state, like Singapore or Monaco, municipal governance from a legal standpoint varies on two dimensions that are keys to the effective authority of governors to manage local affairs: the legal powers of the city, and its geographic boundaries. In traditional legal thinking, nations are the primary unit of sovereignty, though in federal systems provinces or states retain some formal sovereignty under domestic (but generally not international) law. Local governments are mere creatures of the national or state sovereign, and so city authority is defined by provincial or national law, which assigns powers and duties. Municipal authority varies between ‘home rule’, in which cities are delegated the full authority of the higher government, and models in which city powers are stated in specific, limited terms. These reflect national or provincial policies of centralization and decentralization, that may change over time and reflect prevailing beliefs about efficiency and good government—and politics (Wilson, 2000; Plaček *et al.*, 2020).

Although cities with extensive home rule powers can make their own law in a broad range of local matters, they are still subject to a general limitation: in most or all legal systems, even the most powerful cities cannot enact measures that conflict with provincial or national law. This makes national and provincial law an important part of the politics of local governance, in several ways. First, even powerful cities are not free to cross lines of policy set by higher levels of government. For example, although it has broad powers to protect public health, the New York City Board of Health was not allowed to impose a ban on large soda portions in part because, New York’s high court ruled, the legislature had repeatedly declined to regulate in that area (Matter of New York Statewide Coalition of Hispanic Chambers of Commerce v New York City Dept. of Health & Mental Hygiene, 2014). Second, allocation of authority among different governments creates the opportunity for political ‘forum shifting’ by any entity or individual seeking a particular policy outcome (Braithwaite and Drahos, 2000). If, for example, local advocates succeed in getting an urban government to enact a healthy law, such as a limit on smoking or an increase in the minimum wage, opponents may seek provincial or national legislation that supersedes the local law or takes away lawmaking authority on tobacco or minimum wage from the local government (Pomeranz *et al.*, 2019).

Law also sets the geographic boundaries of local legal entities. Law may establish extensive and easily expanded boundaries for a municipal government, so that the city can grow as a spatial legal entity to keep pace with population spread. Or legal boundaries may be smaller than those of the urban area itself, and hard to change, so that one ‘city’ from an economic and social point of view may be (and often is) comprised of many municipalities with more or less discretion to cooperate or compete. Law may compel or offer the option of regional cooperation on one or more functions, like public transport, waste and public amenities like parks (Campos-Alba *et al.*, 2020).

The literature on public management does not provide strong or universal guidance on the optimum organization of local government (Feiock and Scholz, 2010; Plaček *et al.*, 2020), and in any case the complexity and time-lines of government reorganization render that ultimate avenue of governance reform a poor target for health promotion activities. Rather, in every urban center, proponents of health promotion will be faced with the challenge and opportunity of working within existing formal governance structures to have as much impact as possible. And in this effort, law is a useful tool. We discuss examples and strategies next.

## MAKING LOCAL GOVERNANCE HAPPEN—THE PLACE OF LAWS FOR BETTER HEALTH GOVERNANCE

Regardless of the form of government, cities are likely to have legal levers they can manipulate for health promotion. We have already addressed familiar uses of law as a health promotion tool through the regulation behavior like smoking and drinking. But, law typically allows much more. Local governments often also have the capacity through law to enlist local institutions to reinforce behavioral rules and norms, whether the primary rules are in national, provincial or local law. Schools and universities, health care facilities and workplaces can be required by local law to create their own internal policies that limit unhealthy behavior like smoking and encourage healthy behavior like regular exercise or breast-feeding (Cislaghi and Heise, 2019).

Cities that have independent tax authority can use it to influence the price of unhealthy products like sugar sweetened beverages, or to encourage consumption of healthy foods. Along with tax incentives, zoning/land use rules can be used to promote fresh-food markets and reduce the prevalence of junk food. Tax revenues can be directed to the creation of local health promotion foundations, which can be a valuable component of an effective system of local health promotion (Schang *et al.*, 2012). Cities are also typically substantial purchasers of products and services, and the processes and standards for purchasing are or can be set out in legal rules or city operating policies. These can be modified to require healthy foods in city-owned or managed food service operations and vending machines, and in city-owned venues like stadiums, schools, hospitals or concert hall (CityHealth, 2020).

Cities can use their legal powers to address incidental legal effects of policies that they themselves cannot control. Cities generally have limited legal and practical capacity to regulate air pollution directly, but most cities can use planning, land-use and traffic authority to reduce the use of polluting vehicles in the city and encourage options like public transportation and biking. Another good example is drug policy and criminal justice. Where cities are responsible for policing, city leaders can promote greater cooperation between criminal justice and health agencies, change the orientation of policing in healthier directions and create space for harm reduction interventions like syringe exchange and safe drug consumption sites. Cities can increase the role of local communities in security, and support greater police accountability (Wood *et al.*, 2015).

Cities may also have the authority to use law to address deeper determinants of health. The overall level of income or wealth inequality in a country reflects factors well-beyond a local government’s control, but city government nonetheless has levers to directly and indirectly reduce economic and social inequality and their effects. Cities may have the authority to set a minimum wage, or to provide tax relief based on income (Bhatia and Katz, 2001). Cities can use their funds to invest in affordable, safe housing and use land-use and tax laws to encourage private developers to do so. Simply maintaining a working system for land-titling (formalization of ownership) so that low-income people can build capital may be within city control (Galiani and Schargrodsky, 2004; Gandelman, 2010). Cities receiving block funding from the provincial or national level may have the discretion to use the funds in ways that ultimately redistribute resources more equitably. Likewise, local governments typically have primary control over poor people’s entry into the local economy, through mechanisms like shop permits, taxi licenses and ability to sell goods in local marketplaces. Reducing the complexity and cost of participating in legal economic activity can promote wealth accumulation and increase the local tax base (de Soto, 2000). Working conditions and terms of employment may also be susceptible to local control. Cities may be able to mandate paid sick leave, and use regulatory authority over the operation of at least some categories of local businesses to force changes to or shut down businesses that operate unsafely (Gaydos *et al.*, 2011).

Although cities have little capacity to change the larger legal framework of governance or expand their own boundaries, their existing legal authority gives them many tools for making government and governance work better. Essential governance standards of transparency, accountability and honesty are no less critical for being difficult to achieve and maintain. Law can promote standards and practices that help move a local system towards better governance.

Good government starts with internal management and composition of government. Any government is only as honest and effective as its workforce. There are no simple off-the-shelf solutions to problems of housing, transportation, sanitation and education, so the qualifications and motives of those assigned to address these wicked problems looms large as a factor in successful public policy (Lewis, 2018). A local civil service can be a cadre of well-qualified, well-resourced and highly motivated professionals committed to helping the community adapt in a healthy way to the challenges the world presents—or it can be a collection of time-serving patronage hacks with more commitment to party than city. Adherence to—and often reform—of civil service rules is a foundation of good governance (Kamarck, 2017).

A second foundation is the legal requirement of transparency supported by workable procedures and mechanisms. The publication of laws, rules, interpretive guidance, budgets, expenditures and policies is something that local governments can require of themselves by law. In broad terms, ‘open government’ includes not just robust transparency and public access to information, but also activities and processes like government data hackathons, open contracting, participatory budgeting and protections for whistleblowers across the whole of local government (OECD, 2016). Vigorous public participation is both a mechanism of transparency and accountability and, from a governance perspective an adaptive benefit in itself that works through increasing the diversity of perspectives and knowledge in decision-making (Burris *et al.*, 2005).

City bureaucrats and leaders who want to engage the public in thinking about and making decisions for policy have tools they can use and that, in a few places, have been made into legally required practices. Participatory budgeting and health impact assessment have some intuitive appeal, and good results have been reported in particular places (Bhatia and Corburn, 2011), but it is important to recognize that, like most areas of governance, the actual evidence that they improve community health or well-being is weak (Campbell *et al.*, 2018; Waimberg *et al.*, 2018), and there is no reason to believe they cannot also have negative effects (Fernández-Martínez *et al.*, 2020). Furthermore, legal mechanisms may not be necessary and are certainly not sufficient to ensure robust participatory governance (Korfmacher, 2019). Involving stakeholders, especially voices of affected communities, must be core to policy and program development and implementation. This includes deliberate efforts to support the capacity of parliamentarians, regulators, community members and those in the health sector to be effective in their engagement with the policy and legislative processes (Burris *et al.*, 2016a, 2020).

Transparency, accountability and public involvement become even more important in large-scale projects involving huge sums of public money, such as infrastructure projects like water systems, roads and housing. In the global experience, corruption, waste and low-quality products are all too common (Bel, 2020), producing both insalubrious conditions and public apathy and cynicism.

## PROMISING WAYS FORWARD

In advancing urban governance for health and well-being, especially in times of rapid change, a multi-prong strategy is likely to be necessary. Three kinds of action may be particularly beneficial: (i) setting priority areas for intervention likely to improve health, living and working conditions in urban areas, (ii) identifying the co-benefits to be achieved through intersectoral governance, and (iii) enacting health laws to secure a comprehensive framework for assuring good health (World Health Organization, 2016).

The sustainable development goals emphasize the criticality of cross-sectoral collaboration. The WHO suggests that healthy urbanization is one of the areas in which partnership across sectors is likely to achieve significant mutual benefit and health gain. Particular tasks in which laws are important include:

planning for adequate housing, shelter, water and sanitation, electricity and other basic services and upgrade urban slums; assuring access to safe spaces and community settings free from violence, with particular attention to disadvantaged groups; and investing in and regulating for safe and accessible transport, road infrastructure and education to reduce injuries and road traffic accidents.

Additional recommended priority action areas where laws can become essential are: stimulating social development (compulsory education, social protection, labor protection, protection from human trafficking etc.) and protecting health of the environment (e.g. protection from pollution, protection of biodiversity, improving systems for food safety and security etc.; World Health Organization, 2017b). Although many of these measures depend on national level legislation, cities may be able to exercise certain measures within their jurisdictional rights.

Different sectors may collaborate through joint planning and budgeting, community consultation and legislative mandates, but a comprehensive legal framework assures policy coherence and provides a more sustainable approach to system-wide cooperative action. In the era of the SDGs, laws are needed to establish health system governance and stewardship; define and enforce accountability; protect human rights rights; ensure access to affordable, safe and quality health services; prevent and manage public health risk; and promote action on social determinants of health. In health promotion we see an increasing number of countries acting on social determinants of health through requirements for municipal public health and well-being plans and for undertaking health impact assessments on proposed projects and plans (World Health Organization, 2020). In moving forward on these fronts, stakeholder involvement, especially voices of the affected communities, need to be core to policy and program development and implementation. Moreover, WHO points to the importance of supporting the capacity of stakeholders to be effective in their engagement with policy and legislative processes, including parliamentarians, regulators, community members, as well as those who work in the health sector.

## CONCLUSION—IMPROVING URBAN GOVERNANCE FOR HEALTH AND WELLBEING

Research and experience alike validate the proposition that law is an essential tool of public health and good governance. Municipal governments, typically facing national or global challenges with limited resources, can and do manage effectively. Effective public health law work requires the ‘ownership’ of law across the disciplines and organizational silos of public health, including research on health threats and behaviors, policy development, advocacy, implementation/enforcement and evaluation (Burris *et al.*, 2016a,b; World Health Organization, 2020).

Moving forward, several priorities should get due attention and funding. Law as a tool for local health promotion and healthier public policy is just another area in which research has been neglected (Ibrahim *et al.*, 2017). We can and should have more definite evidence sooner about the efficacy of innovations like housing code enforcement, sugar-sweetened beverage taxes and participatory budgeting. Local governments can help by evaluating their own health policies, and by joining with peer governments to collaboratively develop, test, evaluate and tweak legal and governance solutions to common problems (Burris *et al.*, 2020). Sassen *et al*., (2018) writing on cities and social progress, suggest strategic co-governance in areas such as urban planning, revenue and expenditure frameworks, public auditing mechanisms, service-delivery innovation (including digital crowdsourcing), legal frameworks to protect rights, enhancing the culture of public debate and creating innovation labs.

Law and good governance can support all this but are necessary rather than sufficient. A spirit of adaptation and learning in a properly resourced civil service can become a feature of the whole of government and engage the whole of society, including philanthropic and business entities. Vigorous policy experimentalism, with rigorous evaluation, can set the stage for systematic diffusion of effective policies through organizations like the WHO, the Alliance for Healthy Cities and advocacy projects like CityHealth.org. Law, as a scalable and adaptable mode of intervention, can be means of municipal mutual self-help at a time when cities face the world’s most difficult problems.

The adoption, enactment and revision of laws do not happen in the absence of political processes. The 2016 Global Report on Urban Health suggested that a renewed focus on urban governance is the key to assuring health and well-being and closing the health equity gap. Several features of good urban governance were highlighted—participation, civil society and community empowerment, public–private partnership, and intersectoral action (World Health Organization & U. N. Habitat, 2016). Good governance entails mediating between different stakeholder interests by validating the potential health impact of different policy options and enabling stakeholders within and outside of government to take informed decisions. This process of interaction and decision-making among the actors to manage the course of events and address shared problems is central to a dynamic system for governance as well as for the co-creation of health and well-being.

The challenges that city leaders face are typically ‘wicked problems’. Collaborative governance in interdependent network clusters with distributed control and divided authority (Innes and Booher, 2018) may be most likely to create conditions for social learning and problem-solving, thus realizing collective action and conditions for future collaboration. When governance frameworks are in place and working well, there is more assurance that effective laws will be adopted and implemented which address both the health concerns of the community and reflect social norms that prioritizes good health.

## DISCLAIMER

The authors alone are responsible for the views expressed in this article and they do not necessarily represent the views, decisions or policies of the institutions with which they are affiliated.

## References

[daab064-B1] Adams D. (2011) The ‘Wicked Problem’ of planning for housing development. Housing Studies, 26, 951–960.

[daab064-B2] Bel G. (2020) Public versus private water delivery, remunicipalization and water tariffs. Utilities Policy, 62, 100982.

[daab064-B3] Bhatia R. , Corburn J. (2011) Lessons from San Francisco: health impact assessments have advanced political conditions for improving population health. Health Affairs (Project Hope), 30, 2410–2418. 22147870 10.1377/hlthaff.2010.1303

[daab064-B4] Bhatia R. , Katz M. (2001) Estimation of Health Benefits From a Local Living Wage Ordinance. American Journal of Public Health, 91, 1398–1402. 11527770 10.2105/ajph.91.9.1398 PMC1446793

[daab064-B5] Braithwaite J. , Drahos P. (2000) Global Business Regulation. Cambridge: Cambridge University Press.

[daab064-B6] Burris S. , Ashe M., Blanke D., Ibrahim J., Levin D. E., Matthews G. et al (2016) Better health faster: the 5 essential public health law services. Public Health Reports (Washington, D.C.: 1974), 131, 747–753. 10.1177/0033354916667496 PMC5230841 28123219

[daab064-B7] Burris S. , Ashe M., Levin D., Penn M., Larkin M. (2016) A transdisciplinary approach to public health law: the emerging practice of legal epidemiology. Annual Review of Public Health, 37, 135–148. 10.1146/annurev-publhealth-032315-021841 PMC5703193 26667606

[daab064-B8] Burris S. , Blankenship K. M., Donoghoe M., Sherman S., Vernick J. S., Case P. et al (2004) Addressing the “risk environment” for injection drug users: the mysterious case of the missing Cop. The Milbank Quarterly, 82, 125–156. 15016246 10.1111/j.0887-378X.2004.00304.x PMC2690204

[daab064-B9] Burris S. , Drahos P., Shearing C. (2005) Nodal Governance. Australian Journal of Legal Philosophy, 30, 30–58.

[daab064-B10] Burris S. , Hancock T., Lin V., Herzog A. (2006) Emerging Principles of Healthy Urban Governance: A Background Paper for the Knowledge Network on Urban Settings of the WHO Commission on the Social Determinants of Health. World Health Organization Kobe Center, Kobe.

[daab064-B11] Burris S. , Kempa M., Shearing C. (2008) Changes in governance: a cross-disciplinary review of current scholarship. Akron Law Review, 41, 1–66.

[daab064-B12] Burris S. , Korfmacher K., Moran-McCabe K., Prood N., Blankenship K., Corbett A. et al, (2020). Health Equity Through Housing: A Blueprint for Systematic Legal Action. Center for Public Health Law Research, Philadelphia.

[daab064-B13] Burris S. , Wagenaar A. C., Swanson J., Ibrahim J. K., Wood J., Mello M. M. (2010) Making the case for laws that improve health: a framework for public health law research. The Milbank Quarterly, 88, 169–210. 20579282 10.1111/j.1468-0009.2010.00595.x PMC2980343

[daab064-B14] Campbell M. , Escobar O., Fenton C., Craig P. (2018) The impact of participatory budgeting on health and wellbeing: a scoping review of evaluations. BMC Public Health, 18, 822. 29970044 10.1186/s12889-018-5735-8 PMC6029380

[daab064-B15] Campos-Alba C. M. , Prior D., Pérez-López G., Zafra-Gómez J. L. (2020) Long-term cost efficiency of alternative management forms for urban public transport from the public sector perspective. Transport Policy, 88, 16–23.

[daab064-B16] Cislaghi B. , Heise L. (2019) Using social norms theory for health promotion in low-income countries. Health Promotion International, 34, 616–623. 29579194 10.1093/heapro/day017 PMC6662293

[daab064-B17] CityHealth. (2020). Healthy Food Procurement in American Cities: Leveraging Local Purchasing Power to Fight Obesity. https://www.cityhealth.org/healthy-food-procurement (28 April 2021, date last accessed).

[daab064-B18] Commission on Social Determinants of Health. (2008). Closing the Gap in a Generation: Health Equity Through Action on the Social Determinants of Health. World Health Organization, Geneva. 10.1016/S0140-6736(08)61690-6 18994664

[daab064-B19] de Soto H. (2000) The Mystery of Capital: Why Capitalism Triumphs in the West and Fails Everywhere Else. Basic Books, New York.

[daab064-B20] Feiock R. C. , Scholz J. T. (2010) Self-Organizing Federalism: Collaborative Mechanisms to Mitigate Institutional Collective Action Dilemmas. Cambridge University Press, Cambridge, UK; New York.

[daab064-B21] Fernández-Martínez J. L. , García-Espín P., Jiménez-Sánchez M. (2020) Participatory frustration: the unintended cultural effect of local democratic innovations. Administration & Society, 52, 718–748.

[daab064-B22] Galiani S. , Schargrodsky E. (2004) Effects of land titling on child health. Economics and Human Biology, 2, 353–372. 15576243 10.1016/j.ehb.2004.10.003

[daab064-B23] Gandelman N. (2010) Property rights and chronic diseases: evidence from a natural experiment in Montevideo, Uruguay 1990-2006. Research Support, non-U.S. Economics & Human Biology, 8, 159–167. 20627736 10.1016/j.ehb.2010.05.005

[daab064-B24] Gaydos M. , Bhatia R., Morales A., Lee P. T., Liu S. S., Chang C. et al (2011) Promoting health and safety in San Francisco’s Chinatown restaurants: findings and lessons learned from a pilot observational checklist. Public Health Reports, 126, 62–69. 21836739 10.1177/00333549111260S311 PMC3150131

[daab064-B25] Gostin L. O. , Monahan J. T., Kaldor J., DeBartolo M., Friedman E. A., Gottschalk K. et al (2019) The legal determinants of health: harnessing the power of law for global health and sustainable development. The Lancet, 393, 1857–1910. 10.1016/S0140-6736(19)30233-8 PMC7159296 31053306

[daab064-B26] Gostin L. O. , Wiley L. F. (2016) Public Health Law: Power, Duty, Restraint, 3rd edition. University of California Press, Berkeley.

[daab064-B27] Hein W. (2003) Global health governance and national health policies in developing countries: conflicts and cooperation at the interfaces. In Hein W., Kohlmorgan L. (eds), Globalization, Global Health Governance and National Health Policies in Developing Countries: An Exploration into the Dynamics of Interfaces. Deutsche Uebersee Institut, Hamburg, pp. 33–71.

[daab064-B28] Ibrahim J. K. , Sorensen A. A., Grunwald H., Burris S. (2017) Supporting a culture of evidence-based policy: federal funding for public health law evaluation research, 1985-2014. Journal of Public Health Management and Practice, 23, 658–666. 28538338 10.1097/PHH.0000000000000598

[daab064-B29] Innes J. E. , Booher D. E. (2018) Planning With Complexity: An Introduction to Collaborative Rationality for Public Policy. Routledge, London.

[daab064-B30] Kamarck E. C. (2017) A Government- Wide Reform Agenda for the Next Administration. In O’Hanlon M. E. (ed.), Brookings Big Ideas for America. Brookings Institution, Washington, DC, pp. 60–74.

[daab064-B31] Kickbusch I. , Allen L., Franz C. (2016) The commercial determinants of health. The Lancet. Global Health, 4, e895–e896. 27855860 10.1016/S2214-109X(16)30217-0

[daab064-B32] Kjellstrom T. , Mercado S., Sattherthwaite D., McGranahan G., Friel S., Havemann K. (2007) Our Cities, Our Health, Our Future: Acting on Social Determinants for Health Equity in Urban Settings. WHO Kobe Center, Kobe, Japan.

[daab064-B33] Komro K. A. , Livingston M. D., Markowitz S., Wagenaar A. C. (2016) The effect of an increased minimum wage on infant mortality and birth weight. American Journal of Public Health, 106, 1514–1516. 27310355 10.2105/AJPH.2016.303268 PMC4940666

[daab064-B34] Korfmacher K. (2019) Bridging Silos: Collaborating for Health and Justice in Urban Communities. MIT Press, Cambridge, MA.

[daab064-B35] Latt S. M. , Milner A., Kavanagh A. (2019) Abortion laws reform may reduce maternal mortality: an ecological study in 162 countries. BMC Women’s Health, 19, 1. 30611257 10.1186/s12905-018-0705-y PMC6321671

[daab064-B36] Lewis M. (2018) The Fifth Risk: Undoing Democracy. W.W. Nortion and Company, New York.

[daab064-B37] Lin V. (ed.) (2013) *Action on Social Determinants of Health: Case Studies from Australia*. Please provide publisher’s name and location for reference Lin (2013).]

[daab064-B38] Lin V. , Kickbusch I. (eds) (2017) Progressing the Sustainable Development Goals through Health in All Policies: Case Studies from around the World Adelaide. Department for Health and Ageing, South Australia.

[daab064-B39] Markowitz S. , Komro K. A., Livingston M. D., Lenhart O., Wagenaar A. C. (2017) Effects of state-level Earned Income Tax Credit laws in the U.S. on maternal health behaviors and infant health outcomes. Social Science & Medicine (1982), 194, 67–75. 29073507 10.1016/j.socscimed.2017.10.016 PMC5696026

[daab064-B40] Matter of New York Statewide Coalition of Hispanic Chambers of Commerce v New York City Dept. of Health & Mental Hygiene. (2014) 23 NY3d 681.

[daab064-B41] McKee M. , Stuckler D. (2018) Revisiting the corporate and commercial determinants of health. American Journal of Public Health, 108, 1167–1170. 30024808 10.2105/AJPH.2018.304510 PMC6085040

[daab064-B42] McQueen D. V. , Wismar M., Lin V., Jones C. M., Davies M. (eds) (2012) Intersectoral Governance for Health in All Policies: Structures, Actions and Experiences. The European Observatory on Health Systems and Policies, Copenhagen.

[daab064-B43] Montez J. K. (2017) Deregulation, devolution, and state preemption laws’ impact on US mortality trends. American Journal of Public Health, 107, 1749–1750. 29019768 10.2105/AJPH.2017.304080 PMC5637688

[daab064-B44] Montez J. K. , Beckfield J., Cooney J. K., Grumbach J. M., Hayward M. D., Koytak H. Z. et al (2020) US state policies, politics, and life expectancy. The Milbank Quarterly, 98, 668–699. 32748998 10.1111/1468-0009.12469 PMC7482386

[daab064-B45] Montez J. K. , Hayward M. D., Zajacova A. (2019) Educational disparities in adult health: U.S. states as institutional actors on the association. Sociological Research for a Dynamic World, 5, 237802311983534. 10.1177/2378023119835345 PMC6640858 31328170

[daab064-B46] Muchomba F. M. , Wang J. S., Agosta L. M. (2014) Women's land ownership and risk of HIV infection in Kenya. Social Science & Medicine, 114, 97–102. 24922606 10.1016/j.socscimed.2014.05.055

[daab064-B47] OECD. (2016). Open Government: The Global Context and the Way Forward. OECD, Paris.

[daab064-B48] Plaček M. , Ochrana F., Půček M., Nemec J. (2020) Fiscal Decentralization Reforms: The Impact on the Efficiency of Local Governments in Central and Eastern Europe. Springer, Cham, Switzerland.

[daab064-B49] Pomeranz J. L. , Zellers L., Bare M., Sullivan P. A., Pertschuk M. (2019) State preemption: threat to democracy, essential regulation, and public health. American Journal of Public Health, 109, 251–252. 30649941 10.2105/AJPH.2018.304861 PMC6336069

[daab064-B50] Public Health and Wellbeing Act 2008. (2008) Victorian Legislation (Australia), Public Law No. 46 of 2008.

[daab064-B51] Rittel H. W. J. , Webber M. M. (1973) Dilemmas in a general theory of planning. Policy Sciences, 4, 155–169.

[daab064-B52] Sassen S. , Pieterse E., Bhan G., Hirsh M., Falú A., Ichikawa H. et al (2018) Cities and social progress. In IPSP (ed), Rethinking Society for the 21st Century: Report of the International Panel on Social Progress: Volume 1: Socio-Economic Transformations, Vol. 1. Cambridge University Press, Cambridge, pp. 187–224.

[daab064-B53] Schang L. K. , Czabanowska K. M., Lin V. (2012) Securing funds for health promotion: lessons from health promotion foundations based on experiences from Austria, Australia, Germany, Hungary and Switzerland. Health Promotion International, 27, 295–305. 21467099 10.1093/heapro/dar023

[daab064-B54] Stryker R. (2013) Law and Society Approaches. In Wagenaar A. and Burris S. (eds), Public Health Law Research: Theory and Methods . John Wiley & Sons, San Francisco, pp. 87–108.

[daab064-B55] United Nations Department of Economic and Social Affairs Population Division. (2019) World Urbanization Prospects: The 2018 Revision. United Nations, New York.

[daab064-B56] Van Dyke M. E. , Komro K. A., Shah M. P., Livingston M. D., Kramer M. R. (2018) State-level minimum wage and heart disease death rates in the United States, 1980–2015: a novel application of marginal structural modeling. Preventive Medicine, 112, 97–103. 29625130 10.1016/j.ypmed.2018.04.009 PMC5970990

[daab064-B57] Waimberg J. , Cloud L. K., Campbell A. T., Lindberg R., Pollack K. M. (2018) Tracking state-level health impact assessment legislation from 2012-2016. Chronicles of Health Impact Assessment, 3, 1–10.

[daab064-B58] Wilson R. (2000) Understanding local governance: an international perspective. Revista de Administração de Empresas, 40, 51–63.

[daab064-B59] Wood J. D. , Beierschmitt L. (2014) Beyond police crisis intervention: moving “upstream” to manage cases and places of behavioral health vulnerability. International Journal of Law and Psychiatry, 37, 439–447. 24629568 10.1016/j.ijlp.2014.02.016 PMC4142086

[daab064-B60] Wood J. D. , Taylor C. J., Groff E. R., Ratcliffe J. H. (2015) Aligning policing and public health promotion: insights from the world of foot patrol. Police Practice and Research, 16, 211-223. 10.1080/15614263.2013.846982 PMC4465110 26085825

[daab064-B61] World Health Organization. (2016). Regional Framework for Urban in the Western Pacific 2016-2020: Healthy and Resilient Cities. World Health Organization, Geneva.

[daab064-B62] World Health Organization. (2017a). Promoting Health in the SDGs: Report on the 9th Global Conference for Health Promotion. World Health Organization, Geneva.

[daab064-B63] World Health Organization. (2017b). Regional Action Agenda on Achieving the Sustainable Development Goals in the Western Pacific Region. World Health Organization, Geneva.

[daab064-B64] World Health Organization. (2020). Better Laws for Better Health: Western Pacific Regional Action Agenda on Strengthening Legal Frameworks for Health in the Sustainable Development Goals. World Health Organization, Geneva.

[daab064-B65] World Health Organization, & U. N. Habitat. (2016). Global Report on Urban Health: Equitable Healthier Cities for Sustainable Development. World Health Organization, Geneva.

